# Monocyte Distribution Width (MDW) as novel inflammatory marker with prognostic significance in COVID-19 patients

**DOI:** 10.1038/s41598-021-92236-6

**Published:** 2021-06-16

**Authors:** Giovanni Riva, Sara Castellano, Vincenzo Nasillo, Anna Maria Ottomano, Giuliano Bergonzini, Ambra Paolini, Beatrice Lusenti, Jovana Milić, Sara De Biasi, Lara Gibellini, Andrea Cossarizza, Stefano Busani, Massimo Girardis, Giovanni Guaraldi, Cristina Mussini, Rossella Manfredini, Mario Luppi, Enrico Tagliafico, Tommaso Trenti

**Affiliations:** 1 Diagnostic Hematology and Clinical Genomics Laboratory, Department of Laboratory Medicine and Pathology, AUSL/AOU Policlinico, Via del Pozzo 71, 41124 Modena, Italy; 2 Department of Medical and Surgical Sciences, University of Modena and Reggio Emilia, Modena, Italy; 3 Department of Medical and Surgical Sciences, University of Modena and Reggio Emilia, Hematology Unit, AOU Policlinico, Modena, Italy; 4 Department of Surgical, Medical, Dental and Morphological Sciences, University of Modena and Reggio Emilia, Modena, Italy; 5 Department of Anesthesia and Intensive Care, University of Modena and Reggio Emilia, Intensive Care Unit, AOU Policlinico, Modena, Italy; 6 Department of Surgical, Medical, Dental and Morphological Sciences, University of Modena and Reggio Emilia, Infectious Diseases Clinics, AOU Policlinico, Modena, Italy; 7 Department of Life Sciences, University of Modena and Reggio Emilia, Centre for Regenerative Medicine “Stefano Ferrari”, Modena, Italy

**Keywords:** Immunology, Microbiology, Biomarkers

## Abstract

Monocyte Distribution Width (MDW), a new cytometric parameter correlating with cytomorphologic changes occurring upon massive monocyte activation, has recently emerged as promising early biomarker of sepsis. Similar to sepsis, monocyte/macrophage subsets are considered key mediators of the life-threatening hyper-inflammatory disorder characterizing severe COVID-19. In this study, we longitudinally analyzed MDW values in a cohort of 87 COVID-19 patients consecutively admitted to our hospital, showing significant correlations between MDW and common inflammatory markers, namely CRP (*p* < 0.001), fibrinogen (*p* < 0.001) and ferritin (*p* < 0.01). Moreover, high MDW values resulted to be prognostically associated with fatal outcome in COVID-19 patients (AUC = 0.76, 95% CI: 0.66–0.87, sensitivity 0.75, specificity 0.70, MDW threshold 26.4; RR = 4.91, 95% CI: 1.73–13.96; OR = 7.14, 95% CI: 2.06–24.71). This pilot study shows that MDW can be useful in the monitoring of COVID-19 patients, as this innovative hematologic biomarker is: (1) easy to obtain, (2) directly related to the activation state of a fundamental inflammatory cell subset (i.e. monocytes, pivotal in both cytokine storm and sepsis immunopathogenesis), (3) well correlated with clinical severity of COVID-19-associated inflammatory disorder, and, in turn, (4) endowed with relevant prognostic significance. Additional studies are needed to define further the clinical impact of MDW testing in the management of COVID-19 patients.

## Introduction

In the clinical management of SARS-CoV-2-associated disease (COVID-19), inflammatory markers with prognostic value, possibly able to correlate with the evolution of abnormal host response observed in severe cases, can help to improve patients’ monitoring and support therapeutic interventions, in particular when using immunomodulatory treatments. According to the current view, the monocyte/macrophage population is deeply involved in the immunopathogenesis of both systemic and organ (lung) hyper-inflammatory manifestations of severe COVID-19^1–3^. Of note, recent flow cytometry-based studies showed that morphological and inflammation-related immunophenotypic changes in peripheral blood monocytes –i.e. expansion of nonclassical (CD14−, CD16+) and intermediate (CD14+, CD16+) monocyte subsets– may correlate with COVID-19 severity and clinical outcome^4,5^. In addition, metabolic dysfunctions of monocytes with proinflammatory phenotype were disclosed in patients with COVID-19 pneumonia^6^. Moreover, single-cell analyses confirmed abnormal activation of proinflammatory monocytes/macrophages in the peripheral blood, as well as in the bronchoalveolar lavage fluids, collected from patients with severe COVID-19^7,8^.

Monocyte Distribution Width (MDW) is a novel cytometry-based parameter, related to volume modifications in circulating monocytes upon activation. MDW value can automatically be provided by last-generation DxH hematology analyzers (Beckman Coulter, Inc.), along with routine complete blood cell (CBC) count^9–12^. During the last few years, MDW has been demonstrated to constitute a valuable *early sepsis indicator* (ESId test), allowing to rapidly suspect the occurrence of sepsis in patients admitted either to Emergency Department (ED)^9–11^, or to Infectious Diseases Unit^12^. In brief, in these studies, the area under the curve (AUC) for ESId test ranges 0.73–0.87, with remarkable negative predictive values (NPV, 93–97%)^9–12^.

In the latest months, severe COVID-19 immunopathogenesis and its life-threatening clinical features, characterized by hyperinflammation (cytokine storm), T-cell deficiencies and coagulopathy, have been proposed to constitute an emerging paradigm of *viral sepsis*^13–17^. Eventually, such conceptual framing may support the rationale for therapeutic modulation of host immune responses to SARS-CoV-2 infection, in order to promote effective antiviral functions, leading to virus control and elimination, without exaggerated systemic inflammatory response.

By considering these premises, we planned to longitudinally evaluate MDW dynamics during clinical evolution of COVID-19 cases, aiming to obtain information on putative MDW associations with other inflammatory markers, disease courses and final outcome. Hence, in a cohort of COVID-19 patients consecutively admitted to the dedicated intensive/semi-intensive units at our hospital (Modena, Italy) in April-June 2020, we periodically performed MDW testing (basically, whenever a CBC count was required by clinicians). Then, we retrospectively analyzed clinical-laboratory data available for each patient. Here, we provide first data disclosing the potentials of this novel hematologic parameter as prognostic inflammatory biomarker in COVID-19 patients.

## Methods

This is a retrospective, observational, single-center cohort study. A total of 87 consecutive COVID-19 patients (Table 1), admitted to either Infectious Disease (n = 68) or Intensive Care (n = 19) Units in Modena (Italy) within a 6-week timeframe (April-June 2020), also included in the cohort described in a recent study^18^, were enrolled in this non-interventional “real-world” pilot study, aimed to disclose possible relevance of MDW monitoring in COVID-19 patents. For each patient, we recorded demographic data and full medical history, including main clinical features and laboratory results. Of note, about half of the patients received immunosuppressive treatment with anti-IL-6 monoclonal antibody (tocilizumab), according to local therapeutic protocols^18^. This study was approved by the local Ethical Committee (Area Vasta Emilia Romagna, protocol number 177/2020, March 10, 2020). Written informed consent was provided by each patient (or legal representative). All clinical investigations have been conducted according to the Declaration of Helsinki principles. In line with non-interventional design of the study, MDW assessment was performed whenever a CBC count was requested on clinical indication (at the time of admission, then every 1–3 days), and the results were not available to clinicians (thus, no clinical decisions were made on the basis of MDW values).

**Table 1:** Demographics and clinical information of study population.

Characteristics	Values
No. of patients	87
Age, mean (SD) [range], years	66 (16) [16–97]
**Sex**	
Men	46
Women	41
**Outcome, No. (%)**	
Survival	71 (81.6)
Death	16 (18.4)
**Ward of hospitalization, No. (%)**	
Infectious diseases unit	68 (78.2)
Intensive care unit	19 (21.8)
**Treatments, No. (%)**	
Tocilizumab	42 (48.3)
Steroids	25 (28.7)
O2-therapy	55 (63.2)
Non-invasive ventilation (NIV)	26 (29.9)
Invasive mechanical ventilation	17 (19.5)

Whole blood venous samples were collected on K2 EDTA and analyzed on a *UniCel DxH900 Hematology Analyzer* (Beckman Coulter, Inc., CA, USA), according to routine methods for CBC count and determination of positional cell parameters (including MDW). This instrument is able to promptly provide, alongside differential counts of circulating leukocytes, an innovative parameter mathematically defined as ‘Monocyte volume Distribution Width’ (MDW), which is based on unique instrumental capability to measure specific cell volume parameters and calculate standard deviation (SD) of volume distribution within monocyte population, as previously reported in details^10,12^.

The following statistical analyses were applied, by using open-source R statistical software packages (https://www.r-project.org) and GraphPad Prism 8.0.2 (GraphPad Software, CA, USA), in order to correlate MDW values with routine laboratory parameters, clinical trajectories and final outcomes.

**Figure 1:**
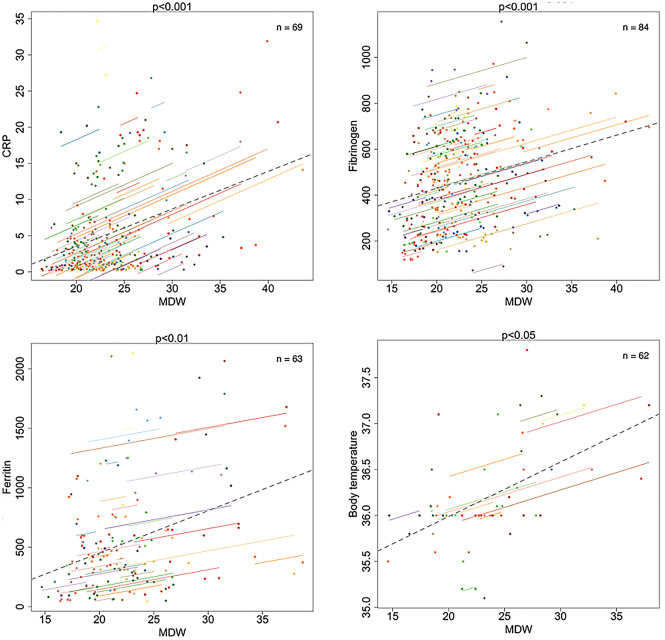
MDW correlations with inflammatory biomarkers in COVID-19 patients. Panels show graphic results of correlation analyses (rmcorr) of MDW with CRP (mg/dL), Fibrinogen (mg/dL), Ferritin (ng/ml) and body temperature (°C). For each plot, *p* values and number of patients (n) tested with each biomarker, are reported. For each patient, paired data (dots) and corresponding regression line are shown with the same color.

Repeated measures correlation (rmcorr) test, implemented in the R package rmcorr (version 0.3.1), provides a coefficient representing the strength of the linear association between two parameters. This statistical method is based on analysis of covariance (ANCOVA) to adjust for inter-individual variability, thus accounting for non-independence among observations. Here we applied rmcorr test to evaluate the correlation between MDW and main inflammatory markers, based on data consisting of repeated measures over time in each patient. In this analysis, only temporally associated measurements (i.e., executed in the same day) were considered (Fig. 1).

**Figure 2:**
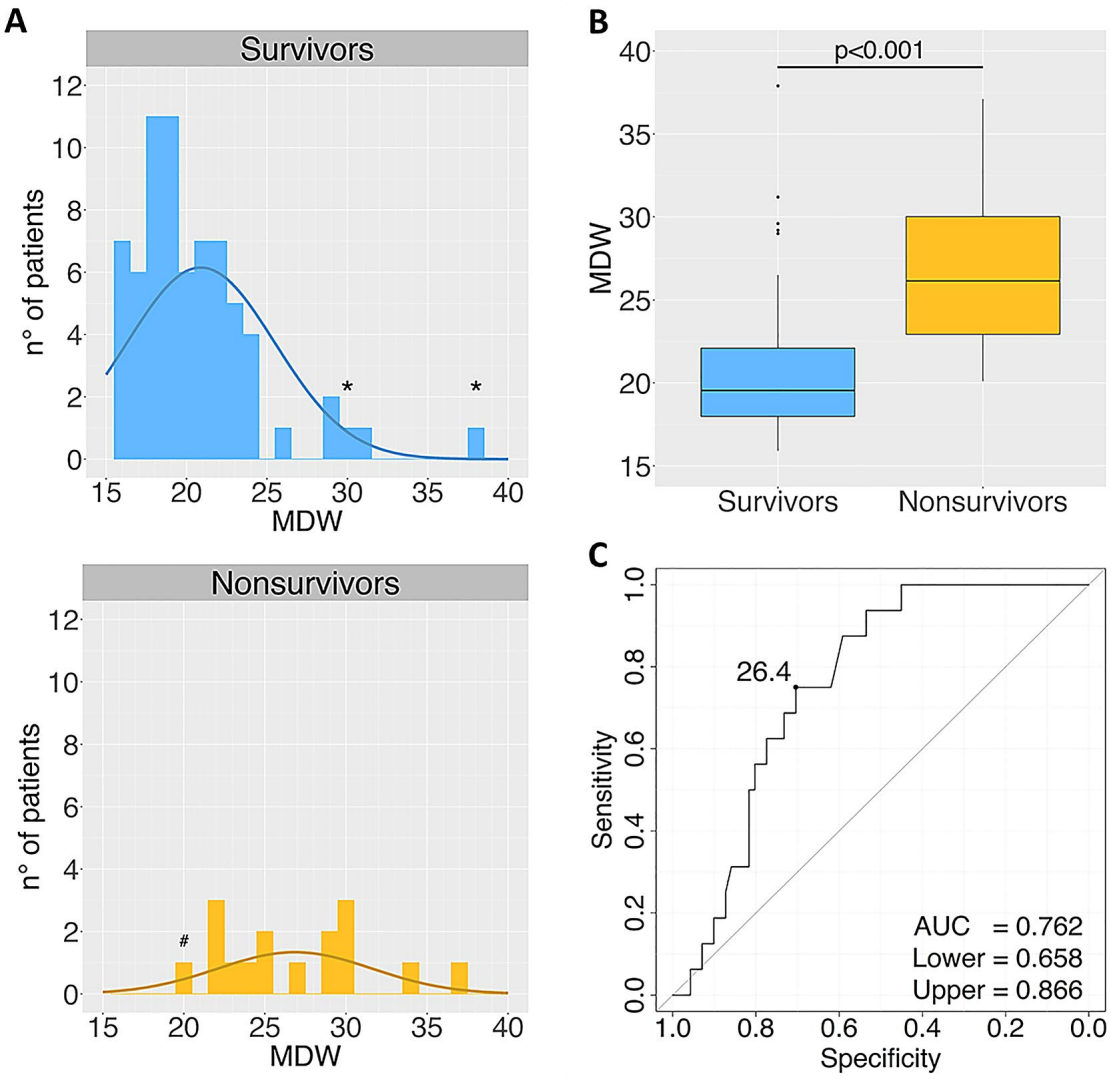
MDW correlations with clinical outcome in COVID-19 patients. (**A**) Distribution of last MDW values detected in COVID-19 patients (n = 87), segregated into different outcome groups (discharged/alive *vs* deceased patients, upper and lower graphs, respectively), as defined at time of censoring. In some cases (*), MDW measurement was lastly performed during critical phases, either in patients with ongoing severe disease (alive at time of censoring), or in patients with a subsequent full recovery but, unfortunately, without any further MDW measurements available. Vice versa, a negative MDW value (< 20.5) was lastly recorded in a patient (#) with complete recovery from hyper-inflammatory pneumonia, who suddenly had fatal event (stroke). (**B**) Boxplot comparison for ‘last MDW’ median values between survivor and non-survivor groups. (**C**) ROC curve for final outcome in COVID-19 patients (n = 87) using MDW test (threshold, 26.4).

The Mann‐Whitney U test, also known as the Wilcoxon rank sum test, tests for differences between two groups on a single, ordinal variable with no specific distribution. By using the R package stats (version 4.0.0), here we applied this test to assess the difference, in terms of ‘last MDW value’ detected in each patient, between survivors and non-survivors (Fig. 2A,B).

Receiver operating characteristic (ROC) curve analysis is a statistical tool based on the notions of specificity and sensitivity, used for assessing diagnostic tests and predictive models. Here, this approach was applied, by exploiting the pROC R package (version 1.16.2), for the identification of the best MDW cut-off to assess the probability of fatal evolution during disease course, within our cohort of COVID-19 patients (Fig. 2C). For each patient, the ‘maximum MDW value’ detected during monitoring was used.

Mixed-effects ordered logistic regression model is a sub-type of logistic regression where the Y-category is ordered; it is used when the dependent variable has a meaningful order, and more than two categories. Both fixed and random effects are considered. Here such analysis, performed using STATA statistical software (release 13; StataCorp LP, TX, USA), served to investigate the relationship between MDW and clinical severity (the latter represented by the ordinal response variable ‘SOFA score’). Both MDW and SOFA score consist of temporally associated (< 12 h), repeated measures over time in each patient.

## Results

In this study, we overall performed 409 MDW measurements during clinical-laboratory monitoring of 87 hospitalized COVID-19 patients, ranging 1–18 MDW detections/patient (median 4 time-points per patient).

### MDW shows significant correlations with inflammatory markers in COVID-19 patients

First, based on the laboratory dataset available for our cohort of 87 COVID-19 patients with at least one MDW measurement, we performed repeated measures correlation analysis (rmcorr) to compare MDW with main inflammatory markers frequently tested in this setting, as part of routine laboratory monitoring. As shown in Fig. 1, a significant correlation with C-reactive protein (CRP) (n = 69 patients, *p* < 0.001), fibrinogen (n = 84, *p* < 0.001) and ferritin (n = 63, *p* < 0.01) has clearly emerged, but not with other common biomarkers (data not shown), such as lactate dehydrogenase (LDH) (n = 84, *p* = 0.70), D-dimer (n = 35, *p* = 0.18) and interleukine-6 (IL-6) (n = 65, *p* = 0.31). In line with another report^19^, here we found that procalcitonin (PCT) resulted negative (< 0.5 ng/mL) in the majority of the tests performed (135/228), thus showing only a limited impact in the clinical monitoring of COVID-19 patients, and hampering the execution of significant comparisons. However, it could also be the case that PCT may better reflect the development of overcoming severe opportunistic infections (i.e. bacterial sepsis), which can complicate the clinical course of severe COVID-19 patients undergoing long-term hospitalization^20^. Of note, we also observed a good association between MDW values and body temperatures (*p* < 0.05), further indicating that high MDW values may be associated with inflammatory syndromes, often including fever (body temperature > 37 °C).

### MDW is associated with clinical outcome in COVID-19 patients

In our cohort, we recorded 71 (81.6%) favorable cases and 16 (18.4%) fatal outcomes. By applying Mann–Whitney test for independent values, we found a significant correlation (*p* < 0.001) between the last value of MDW detected in each patient (n = 87), and the final clinical outcome (survival/discharge *vs* death). Indeed, by considering the group of 71 surviving patients, the median *last MDW value* was 19.6, while this median rose up to 26.1 in the group of 16 patients with unfavorable outcome (Fig. 2A,B). Such observation prompted us to further investigate whether MDW could provide prognostic information in this setting. Therefore, by performing ROC curve analysis (Fig. 2C), we identified a MDW value of 26.4 as best cut-off to assess the probability of fatal evolution during the disease course in our cohort of COVID-19 patients (n = 87). This analysis provided a promising area under the curve (AUC) of 0.76 (95% CI: 0.66–0.87; sensitivity, 0.75; specificity, 0.70). In details, among 33 out of 87 patients showing at least one MDW value > 26.4, 12 cases had fatal outcome, meaning that high MDW values are associated with a mortality rate (absolute risk) ≈35%. Of note, almost all the remaining 21 patients, who survived despite high MDW levels, also developed a critical phase characterized by a hyper-inflammatory disease (pneumonia), which however was well controlled and, eventually, had a favorable clinical course, often associated with a consistent decline of MDW values. On the other hand, only 4 out of 54 patients (< 10%) died although showing MDW values always lower than 26.4 (Negative Predictive Value, NPV = 0.93). Putting together these data, by applying Yates’ corrected Chi-square test, we detected a significant association (*p* < 0.01) between MDW values and final clinical outcomes, also showing remarkable risks of fatal evolution when MDW values were above 26.4 (RR = 4.91, 95% CI: 1.73–13.96; OR = 7.14, 95% CI: 2.06–24.71; RR and OR *p* values = 0.001).

### MDW may be associated with disease severity and clinical course in COVID-19 patients

In order to evaluate whether MDW values could be associated with different levels of disease severity (putatively driven by detrimental inflammation) during COVID-19 clinical course, we compared MDW values with a common score clinically used to monitor critical patients, i.e. ‘sequential organ failure assessment’ (SOFA score), which was periodically calculated in our COVID-19 patients, basically after each execution of blood gas analysis. To this aim, we were able to identify a group of 16 patients (12 surviving and 4 deceased), showing temporal associations (< 12 h) between MDW measurement and SOFA score assessment, which allowed to perform an insightful statistical analysis, according to the ‘mixed-effects ordered logistic regression model’. Indeed, by using this approach, we revealed a significant direct correlation between MDW and SOFA dynamics (*p* < 0.001). As conceivable, we observed that, in most cases, SOFA score was chiefly determined by the level of respiratory dysfunction; notwithstanding, SOFA score could further increase when additional organ impairments occurred. Interestingly, in those few cases without severe acute respiratory failure, i.e. with good PaO2/FiO2 (> 300–400 mmHg, scoring 0–1 points according to SOFA calculation rules for respiratory function), but characterized by higher (> 4) total SOFA scores, due to other organ impairments (often related to pre-existing comorbidities), we detected low MDW values, as well as low general inflammation levels. Indeed, in this group (n = 16), the correlation between MDW values and the PaO2/FiO2 score alone (calculated according to SOFA rules) resulted to be still significant (*p* < 0.001). These findings may be well in agreement with the primary role proposed for monocyte/macrophage population in the development of inflammatory lung injury, characterizing severe COVID-19 pneumonia^1–3^.

**Figure 3:**
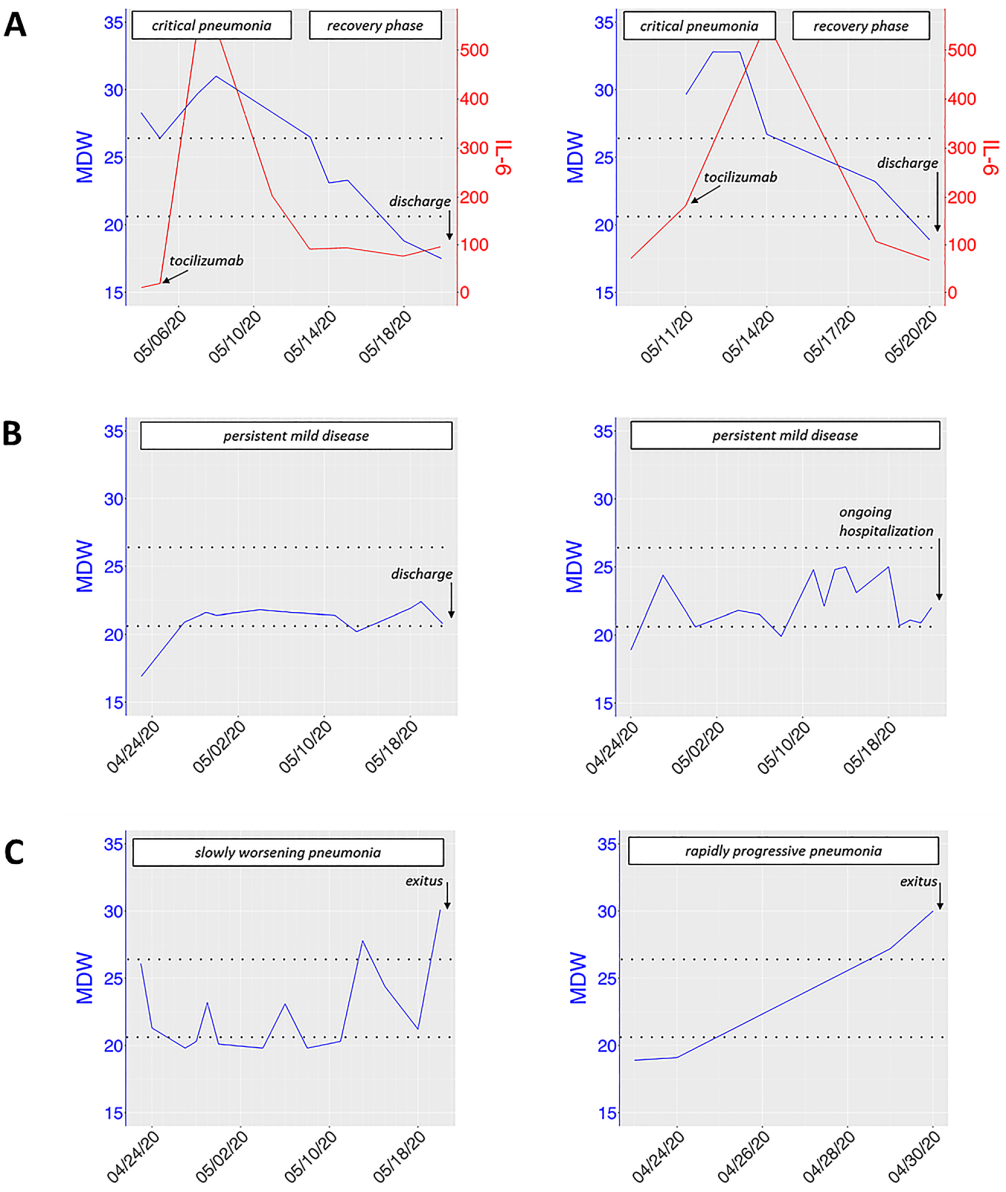
MDW patterns may depict clinical trajectories of COVID-19 patients. (**A**) Exemplificative clinical journeys of two COVID-19 patients with favorable outcome, both showing a critical phase (‘severe COVID-19’), followed by a complete recovery after anti-IL6R therapy (tocilizumab). In these patients, MDW reached high values (> 26.4), then, after immunosuppressive treatment, progressively decreased below negative threshold (< 20.5). MDW, blue line; IL-6 (pg/mL), red line. For MDW pattern comparisons, (**B**) two exemplificative COVID-19 cases without a critical respiratory impairment (thus not treated with tocilizumab), but with prolonged inflammation-driven illness and persistent low/mid-elevation of MDW values (ranging 20.5–26.4), as well as (**C**) two fatal COVID-19 cases, with MDW values progressively growing above 26.4.

Finally, in 21 out of 87 COVID-19 patients, we were able to perform at least 7 serial MDW detections for each case, allowing to uncover interesting patterns of MDW trends and observe some notable associations with COVID-19 clinical evolution (Fig. 3A–C). Of note, in some patients, tocilizumab infusion was followed by complete recovery from a critical phase, characterized by life-threatening respiratory dysfunction (Pa02/FiO2 < 200 mmHg) (Fig. 3A).

These patients obtained a progressive reduction of MDW values, inflammatory signs and SOFA score. However, soon after tocilizumab administration, IL-6 plasma levels showed massive bursts –up to 1 log, for several days– typically induced by the competitive inhibition of IL-6 receptors^21^. This effect can obviously blur the role of IL-6 testing in patients treated with tocilizumab. Besides, MDW patterns well followed the course of the disease, with MDW peak values (> 26.4) detected during the utmost critical phase, while, few days after tocilizumab infusion, MDW values progressively decreased till to negative values (< 20.5). Conversely, both patients still showed abnormal IL-6 levels at time of discharge. In line with these observations, a recent single-cell analysis in two severe COVID-19 patients showed important monocyte subset modifications after tocilizumab therapy^7^. Functional studies are warranted to explore further the connections between MDW, cytokine levels and cytokine-targeted immunomodulatory treatments.

## Discussion

In this study, we first show that MDW represents a novel inflammatory marker, readily exploitable in the clinical-laboratory monitoring of COVID-19 patients, along with cytokines, acute-phase reactants and other components of humoral innate immunity^19,20,22–24^. When compared with these classical inflammatory markers (plasma proteins), MDW basically differs in being a cell-based (monocyte) cytometric parameter. Hence, MDW measurement may provide a novel type of information (i.e. non-humoral) to define the inflammatory status of the patient. Moreover, MDW should not be hindered by possible therapy-related hemodilution, whilst theoretically, it could be not determinable in the case of profound monocytopenia (i.e. monocytes < 100/μL). However, in our experience, COVID-19 patients rarely showed such remarkably low monocyte count, and indeed, MDW measurement resulted undeterminable in < 5% of tests performed.

Moreover, as main finding of this work, well in agreement with the emerging notion that monocyte/macrophage activation can play a pivotal causative role in the inflammatory disorder characterizing severe COVID-19^1–8^, here we show that MDW can serve as novel prognostic biomarker in hospitalized COVID-19 patients. In particular, we found that *high MDW values*, identified by using 26.4 as AUC-ROC threshold, are associated with elevated mortality risk (about one third) in our series of 87 COVID-19 patients. Possibly in line with this finding, in a series of 41 COVID-19 patients tested once for MDW at ED admission, MDW mean value was 28.8 in very critical patients (admitted to ICU after ED triage), while a mean value of 25.4 was observed in patients with milder symptoms^25^. thus supporting the idea that severe clinical conditions can be associated with higher MDW values. In the original studies defining ESId test, based on two wide cohorts of consecutive ED patients^9,10^, MDW values of 20 and 20.5 were identified as best thresholds, respectively, to consider the test as positive. However, MDW is a recent biomarker –with immunobiological implications and clinical significance still partially unknown– and further validation studies are now required to define optimal cut-offs in MDW test.

Additionally, by performing a regression statistical analysis, although limited to a small subgroup of patients (n = 16, accounting for total 170 timepoints tested), we observed that MDW may also correlate with disease severity (SOFA score). Whether MDW changes over time can be predictive of COVID-19 progression from mild manifestations to subsequent organ failures, and in this case, whether MDW test can usefully be adopted by clinicians for guiding early interventions (e.g., closer monitoring, starting immunomodulatory treatments, etc.) are intriguing questions that remain to be assessed by planning ad hoc prospective interventional studies. Interestingly, MDW testing could also contribute to define patients taking advantage of timely treatment with recently-approved SARS-CoV-2-specific monoclonal antibodies.

As basic advantage in clinical practice, this hematologic parameter can directly be provided within routine CBC counts –even without requiring clinicians to specifically ask it– allowing a simple and fast monitoring of the inflammatory state in COVID-19 patients. Our findings indicate that MDW evaluation could worthily be implemented in routine assessment of COVID-19 patients, and, in particular, MDW monitoring may help clinicians both to identify the correct timing for immunosuppressive treatments, and to assess the related response. In addition, also in the case of sudden aggravation of clinical status, MDW could provide a specific and easy-to-obtain hint on the actual severity of patient’s hyper-inflammatory state (cytokine storm). Intriguingly, also in mild COVID-19 cases managed in outpatient settings, MDW could be easily and fruitfully tested as disease severity biomarker to guide patient’s hospitalization and early therapeutic interventions (e.g., specific monoclonal antibodies).

In perspective, new investigations on MDW testing in larger series of patients and in different COVID-19 settings are needed to overcome the limitations of this ‘proof-of-concept’ real-life study, and possibly to extend its results, in particular aiming to: (1) better explore MDW correlations with inflammatory serum biomarkers, as well as with immunological subsets of circulating monocytes, dendritic cells and lymphocytes^26,27^; (2) prospectively evaluate the prognostic roles of MDW in COVID-19 patients, either as ‘single assessment’, or as ‘pattern evaluation’ in MDW dynamics, possibly allowing consistent predictions on short- and long-term clinical evolutions; (3) disclose possible impact of different medications on MDW values; (4) implement the prognostic significance of MDW into multiparametric predictive models. In particular, by considering that MDW test provides peculiar information on actual inflammatory state, MDW evaluation could usefully be included in prognostic models of organ impairment assessment (e.g. SOFA), as indeed recently proposed for sepsis^11^. Moreover, by considering the important prognostic role of T-cell lymphopenia in COVID-19^28^, an innovative parameter combining these two cytometric values (putatively called “MDW-to-lymphocyte ratio”) could worthily be investigated. This suggestion seems consistent with COVID-19 studies highlighting the predictive role of neutrophils-to-lymphocyte ratio (NLR) and other similar cell ratios, based on combination of myeloid and lymphoid cell subsets^29–31^. Very recently, a diagnostic test using NLR and MDW has been demonstrated to accurately distinguish COVID-19 from influenza and common upper respiratory tract infections in patients with suspected symptoms (AUC: 0.84; MDW threshold, ≥ 20)^32^.

While other reports have recently started to describe the performance of MDW as diagnostic marker of COVID-19^25,32^, in this work we focused on MDW use in the monitoring of hospitalized patients diagnosed with COVID-19, and we disclosed, for the first time, the potentials of MDW as prognostic marker during COVID-19 course. Our study suggests that virtually all COVID-19 in-patients present at least one positive value (> 20.5) of MDW, which, indeed, constitutes a novel sepsis biomarker. This fact strongly supports the notion that severe COVID-19 can be considered a new viral sepsis^13–17^, being characterized by cytokine storm and remarkable T-cell lymphopenia, which well parallel severe inflammatory response syndrome (SIRS) and T-cell suppression, typically occurring in bacterial sepsis^33^, as well as in other sepsis of viral origin^34^.

## Conclusions

Our study reveals a promising role for MDW as new inflammatory cytometric marker, with valuable prognostic significance during the monitoring of COVID-19 patients. In particular, MDW may be relevant as severity marker of COVID-19-associated inflammatory disease, as well as may have a role in the assessment of therapeutic response after immunomodulatory treatments. Further studies, performing serial MDW measurement in combination with other inflammatory/sepsis biomarkers, immunophenotypic profiling and clinical severity scores, are warranted to shed new light on immunobiological implications of MDW modifications, and to prospectively evaluate the clinical impact of MDW monitoring in COVID-19 patients.

## Data Availability

The datasets used in this study are not publicly available since they are still under elaboration for publication by the authors, but are available from the corresponding author on reasonable request.
